# S-nitrosylation of cardiac myocyte proteins may underlie sex differences in cardiac disease

**DOI:** 10.3389/fphys.2025.1565917

**Published:** 2025-05-06

**Authors:** Esther U. Asamudo, Raquel E. Parackal, Jeffrey R. Erickson, Donald M. Bers

**Affiliations:** ^1^ Department of Pharmacology, University of California, Davis, Davis, CA, United States; ^2^ Department of Physiology and HeartOtago, University of Otago, Dunedin, New Zealand

**Keywords:** cardiovascular diseases, cardiac proteins, S-nitrosylation, calcium handling, sex differences, excitation-contraction coupling

## Abstract

Nitric oxide (NO) plays several critical roles in cardiovascular physiology. This molecule regulates cardiac function by modifying Ca^2+^-handling proteins through a process known as *S-*nitrosylation. These targets include L-type Calcium Channels (LTCC), Ryanodine Receptors (RyR2), Protein Kinase G (PKG), Phospholamban (PLB), sarco/endoplasmic reticulum Ca^2+^-ATPase (SERCA2a) and Ca^2+^/Calmodulin-dependent protein kinase II (CaMKII). *S-*nitrosylation is a covalent attachment of an NO moiety to the thiol side chain of a cysteine residue within a protein. This process can modify excitation-contraction coupling in cardiomyocytes and may mediate some forms of cardioprotection. Several studies have shown that *S*-nitrosylation may also be involved in the progression of cardiovascular diseases. Most importantly, recent studies have focused on the molecular mechanisms underlying cardiovascular diseases (CVD). Emerging evidence suggests that sex-specific differences in cardiac protein *S-*nitrosylation exist, and may partially explain disparities in cardiovascular health in males and females. Females have been found to have higher cardiac protein *S-*nitrosylation levels compared to men, and this is attributed to enhanced NO production through estrogen. Emerging data suggests that S-nitrosylation of specific proteins such as CaMKII has a dual role of promoting and preventing arrhythmias, it is not clear whether the cardioprotective effect of *S*-nitrosylation of specific cardiac proteins is sex-dependent. A deeper understanding of the mechanisms regulating the role of protein S-nitrosylation and the impact of sex differences on S-nitrosylation will open new avenues for therapeutic interventions in cardiac diseases.

## 1 Introduction

Post translational modifications (PTMs) are known to regulate the functioning of cardiac proteins. For instance, *O*-GlcNAcylation is a PTM that has been described as a sweet-bitter one due to its protective or destructive effect depending on the duration and targets protein *O*-GlcNAcylation (Chatham and Marchase, 2010; Jensen et al., 2013a; Kristiansen et al., 2019; Ng et al., 2021; Okolo et al., 2023; Pælestik et al., 2017; Rao et al., 2004). Similarly, oxidation is a known player in cell health and signaling, where oxidative stress results from an imbalance between production from metabolism and accumulation of reactive oxygen species (ROS) in cells (Münzel et al., 2017; Pizzino et al., 2017). The impact of oxidation on cardiac ryanodine receptors (RyR2), the gatekeeper for bulk calcium ion (Ca^2+^) release in the heart, has been described as biphasic (Belevych et al., 2009; Terentyev et al., 2008; Waddell et al., 2016). At the single-channel level, oxidation RyR2 is known to either activate or inhibit the channel, all depending on the level of oxidation (Waddell et al., 2016). *S-*nitrosylation has also been identified in the modulation of the activity of cardiac proteins such as RyR2, sarco/endoplasmic reticulum Ca^2+^-ATPase (SERCA2a), Ca^2+^/Calmodulin-dependent protein kinase (CaMKII) and L-type calcium channels (LTCC) involved in excitation-contraction coupling (ECC). Another widely known PTM of cardiac proteins that drives cardiac function is phosphorylation. Interestingly, there are over 500 known protein kinases in the human kinome, where a good number of these kinases have been directly implicated in cancer, inflammatory, cardiovascular and metabolic disease progression (Ather et al., 2013; Chen et al., 2022; Cohen, 2001).

One current research focus has been on elucidating the molecular mechanisms that disrupt cardiac ECC, leading to arrhythmias and heart failure. The key players identified in cardiac functioning whose regulation or dysregulation could tip the scales from health to disease. Two of such drivers include PTMs (e.g., phosphorylation, *O-*GlcNAcylation, *S-*nitrosylation and oxidation) and sex differences. Further detail on the roles of these drivers in defining cardiac function, and the inter-relationship between them, will be discussed in this brief review. The term, cardioprotection will be used below and refers to all acute or chronic effects and strategies that aid in preservation of the heart against damaging stresses (Kübler and Haass, 1996). This can be achieved through several adaptive physiological and compensatory strategies or by therapeutic approaches.

With these in mind, this review aims to discuss how NO interacts with CaMKII in the heart and its effect on cardiovascular function as well as offer insights on the interplay between *S-*nitrosylation and sex differences.

### 1.1 Key players in cardiac functioning and cardioprotection

Before diving into molecular mechanisms affecting cardiac function, it is expedient to note that other factors such as genetic, epigenetics, hormonal, neurohumoral, circadian, environmental, molecular, cellular, sex differences and pathological influences, also feed into the cardiovascular regulation loop (Chang et al., 2021; Grandi et al., 2023; Hall et al., 2000; Peliciari-Garcia et al., 2018; Tao et al., 2023; Zhang et al., 2020). In fact, it is known that the PTM of proteins is an important factor responsible for modulating protein function, stability, and localization (Hirano et al., 2016; Lee et al., 2023). Evidence has emerged on the nature of many protein PTMs (phosphorylation, *O*-GlcNAcylation, *S-*nitrosylation, etc.) in tissues and organs such as the heart (Zhang et al., 2020; Young, 2023; Zhang and Jain, 2021). These highlight the intricate relationships between different factors that regulate cardiac function, suggesting a complex role of cardiac proteins in the heart. For instance, the mitochondria has been identified as a central regulator of cellular redox balance and considered a primary source of reactive oxygen species (ROS) in the heart (Shi and Qiu, 2020). The bioactivity and quality control of mitochondrial function are tightly regulated by reversible *S*-nitrosylation and denitrosylation of mitochondrial proteins (Ozawa et al., 2013). In response to changes in mitochondrial respiration and redox state, mitochondrial proteins can undergo *S*-nitrosylation, which serves to protect reactive thiol groups from irreversible oxidation and limits excessive ROS production (Piantadosi, 2012; Fernando et al., 2019). This redox-sensitive modification contributes to cellular defense by preserving mitochondrial integrity, preventing membrane permeabilization, and inhibiting apoptosis (Fernando et al., 2019; Whiteman et al., 2006). Given that mitochondrial dysfunction is a key contributor to cardiac disease, mitochondrial protein *S*-nitrosylation represents an important cardioprotective mechanism in conditions of oxidative stress.

Abnormalities in protein structure and function in any part of the cardiovascular system can impair cardiac function leading to CVDs such as arrhythmias, congenital heart diseases, coronary artery diseases, heart failure and stroke (Chakraborty et al., 2019; Jiang et al., 2004). The involvements of PTMs in exacerbating cardiovascular dysfunction and injuries have been widely studied (Okolo et al., 2023; Chakraborty et al., 2019; Asamudo and Erickson, 2019; Erickson et al., 2008; Erickson et al., 2013). Also known are the roles and influence of some PTMs as cardioprotective factors during disease or injury states such as (a) the reduction of infarct size by ischemic pre-conditioning and cushioning against ischemia/reperfusion (I/R) injury with O-GlcNAcylation, (b) leveraging *S-*nitrosylation to shield the heart from ischemic reperfusion injury and arrhythmias, and (c) as prospective tools for cardiac regeneration therapy (Jensen et al., 2013b; Li et al., 2022; Shiva et al., 2007).

### 1.2 Sex differences in cardiac function

While the component proteins of the heart determine its regulation, sex differences have emerged as another player in cardiovascular health, pre-disposition to and survival from certain diseases (Peters et al., 2019). Sexual dimorphism of the cardiovascular system begins from puberty when circulating sex hormones are stimulated to be produced from the gonads (Cabral et al., 1988). In males, this causes a 15%–30% expansion of heart mass, and expansion not seen in females (Cabral et al., 1988), resulting in a significantly smaller ventricle size and functional end diastolic dimensions in females (Parks and Howlett, 2013). Despite this anatomical difference, it is well established that basal cardiac function in females is hyperdynamic in comparison to males. Cardiac magnetic resonance imaging (MRI) and ventriculography studies have established female hearts to have a significantly higher heart rate, ejection fraction, relaxation rate and a significantly lower end-diastolic volume and end systolic volume (Parks and Howlett, 2013; Chung et al., 2006). The Dallas Heart study established there to be no significant difference in ejection fraction between males and females for a given end systolic volume. However, for any given end diastolic volume, females facilitated a significantly higher stroke volume and thus significantly higher ejection fraction in comparison to males (Chung et al., 2006). Due to the greater ventricle mass, males are able to facilitate a greater increase in ejection fraction than females in exercise conditions (Parks and Howlett, 2013).

Literature abounds on the links between cardiac function and sex-specific hormones which have receptors on cardiomyocytes (Curl et al., 2009; Murphy, 2011). Gonadectomy studies have established testosterone as determinant of the sexual dimorphic differences in cardiac function from a transcriptional level. Testosterone increases expression of Ca^2+^ channels and transporters to facilitate cardiac function characteristics of a male heart. However, gonadectomy resulted in hypo-contractility which was rescued by testosterone supplementation (Curl et al., 2009; Golden et al., 2003). This may explain the stronger response to exercise in males when compared to females. Female ovariectomy studies have established that estrogen has an important role in reducing SR Ca^2+^ stores and dampening Ca^2+^ transients (Fares et al., 2012). Furthermore, the sensitivity of cardiac myofilaments to Ca^2+^ increases significantly in ovariectomy rats, an effect rescued upon estrogen supplementation (Wattanapermpool et al., 2000). It is worth mentioning that progesterone is also able to attenuate Ca^2+^ sensitivity of myofilaments in the cardiomyocytes of female mice, but not male mice (Feridooni et al., 2017).

Echocardiography studies have established diastolic function of young females to be better than age matched males. However, with increasing age, males have worsening systolic function that is not observed in females (Grandi et al., 1992). The age-associated decline in testosterone in males and estrogen in females has been said to be associated with the corresponding age groups of increased risk of CVD prevalence (Kaushik et al., 2010). In males, the decline in testosterone occurs from age 50. However, it is suggested that this may occur years prior and impact cardiovascular function at an insignificantly lower level (Kaushik et al., 2010). In females, the decline in estrogen caused by menopause is well established to correlate with an increase in CVD mortality as discussed previously. However, there is no clear evidence of when the impact on cardiac function begins and whether it is related to insignificant decreases in circulating estrogen. There may also be changes further down the sex hormone transduction pathways of the cardiomyocytes that contribute earlier. That is, subcellular channels and enzymes that may become dysfunctional years prior and contribute more measurably to dysfunction when circulating sex hormones have significantly declined. This kind of subcellular dysfunction could explain why hormone replacement fails to rectify the problem, as the transduction pathway has developed external pathological triggers for signal transduction to occur in the absence of sex hormones. In CVD, it is well established that the autonomic nervous system increases sympathetic stimulation of the heart regardless of whether there is need for it. As sex hormone signaling work synergistically with sympathetic β-adrenergic signaling in the heart, it is plausible that a similar dysfunction is occurring in sex hormone transduction pathways.

### 1.3 Sex differences in cardiovascular disease prevalence

CVD is currently the highest ranked cause of death in globally and was historically considered a ‘disease of men’. However, CVD prevalence is currently higher in females when compared to males (Appelman et al., 2015). The net global burden of CVD is attributable to risk factors such as hypertension, smoking, obesity, diabetes, and hypercholesterolemia. Notably, associated mortality of CVD in males is higher than in females in all age groups, with difference particularly visible in the 40–44 age group (Mikkola et al., 2013). Unlike males, the associated mortality of CVD in females only increases from the 55–59 age group onwards, which is considered clinically as post-menopausal (Mikkola et al., 2013).

A significant contribution of this is caused by heart failure (Kobak et al., 2022; Martin et al., 2025). Two subcategories of heart failure (HF) may reflect sex determined CVD morbidity and associated mortality trends. HF with reduced ejection fraction (HFrEF) is a primarily a ventricular dysfunction, associated mostly by systolic dysfunction following myocardial damage. On the other hand, HF with preserved ejection fraction (HFpEF) is a multi-organ syndrome associated with diastolic dysfunction and affects more females than males (Kobak et al., 2022; Dunlay et al., 2017; Pepine et al., 2020). Prevalence increases significantly from age 55 in both sexes but becomes predominant in post-menopausal females (Pepine et al., 2020; Sotomi et al., 2021). However, HFpEF is now becoming the dominant form of HF associated with the worsening global diabetes and obesity epidemics. The decline of estrogen after menopause may hasten loss of cardioprotective mechanisms signaled by estrogen (Kobak et al., 2022; Pepine et al., 2020; Alex et al., 2018; Shuaishuai et al., 2023). This loss of estrogen is thought to be associated with the higher prevalence of females with obesity, diabetes, hypertension, autoimmune disease, and heightened immune function (Kobak et al., 2022; Pepine et al., 2020). Thus, it has become imperative that CVD risk factors specific to females become elucidated clearly to reduce the post-menopausal mortality risk (Appelman et al., 2015; Mikkola et al., 2013). Investigating this will not only improve diagnosis of females with CVD but also improve clinical outcomes.

The changes in estrogen signaling are not the only key contributors to CVD prevalence. Sex differences have been suggested to underlie key biochemistry and physiology aspects of cardiac function, and that dysfunction could occur prior to age-related decline of sex hormones (Casin and Kohr, 2020). 10% of females over the age of 80 have HFpEF as aging has been established to facilitate molecular and cellular changes involves in the pathophysiology (Kobak et al., 2022; Pepine et al., 2020; Sotomi et al., 2021; Shuaishuai et al., 2023). Redox imbalance caused by reduced antioxidant capacity and significant increase in ROS and RNS (reactive nitrogen species) production is one of the mechanisms that facilitate the aging of the heart (Martín-Fernández and Gredilla, 2016; Pagan et al., 2022). The epigenetic involvement of ROS/RNS-induced aging is associated with excessive *S-*nitrosylation of cardiomyocyte mitochondrial proteins (Hu and Ren, 2016; Schulman and Hare, 2012). A previous study reported a higher number of *S-*nitrosylated proteins in the mitochondria of female cardiomyocytes compared to males (Shao et al., 2016). It was suggested that this may be a key cardioprotective mechanism against irreversible oxidative damage. The same study also reported GSNO-R expression to not be significantly different to male cardiomyocytes. However, the GSNO-R activity in the female whole heart homogenates was significantly higher than the males. This was in accordance with another study (Brown-Steinke et al., 2010) that reported increased GSNO-R activity in female mice lungs compared to males, but no difference in expression. In the context of aging, these studies suggest that estrogen signaling in female hearts may be increasing GSNO-R activity to protect mitochondria from ROS/RNS induced premature aging.

Taken together with the cardioprotection of estrogen signaling, it is plausible that the cardiac antioxidant systems associated with biological males are vulnerable to becoming overwhelmed, predisposing them to premature aging of the heart. This is supported by a recent review (Qian et al., 2024) highlighting three key mechanisms that allow estrogen signaling, via the membrane bound GPR30 estrogen receptor, to prevent systolic and diastolic dysfunction. 1) Estrogen signaling increases the transcription of antioxidant defense against elevated ROS/RNS, 2) estrogen signaling prevents the pro-fibrotic and pro-apoptotic cascades and promotes physiological hypertrophy, and 3) estrogen signaling promotes increased atrial natriuretic peptide production to inhibit the Renin-Angiotensin-Aldosterone System cascade directly. Furthermore, estrogen signaling regulates BH4, a vital enzyme cofactor required for the synthesis of nitric oxide, to prevent the uncoupled eNOS (NOX) RNS production that characterizes atherosclerosis pathophysiology (Arias-Loza et al., 2013).

In the absence of estrogen signaling to upregulate antioxidant systems, biological male redox balance is prone to becoming overwhelmed much earlier in life. This may explain why biological males develop CVD much earlier in females as the features of cardiac aging would arise after estrogen signaling reduces. Thus, the gradual loss of estrogen regulation of antioxidant systems upon menopause is likely to reduce the protection of female hearts from premature aging and therefore promote cardiovascular dysfunction.

Further research into these biochemical and physiological sex differences could inform sex-specific diagnostics to prevent the progression of CVDs. This would not only be imperative to combat the worldwide burden of CVD morbidity and associated mortality but also contribute to improving quality of life by influencing the development of therapeutic interventions that take sex into consideration (Casin and Kohr, 2020).

### 1.4 Protein *S-*nitrosylation and cardiac function

Nitric oxide (NO) is synthesized from L-arginine by nitric oxide synthase (NOS). NOS has three main isoforms; Neuronal NOS (nNOS or NOS1), endothelial NOS (eNOS or NOS3) and inducible nNOS (NOS2; induced in heart after inflammation and is constitutively active). NO is known for its role in vasodilation of the vasculature via the stimulation of soluble guanylyl cyclase (Chen et al., 2008; Moncada et al., 2006) and its control of mitochondrial oxygen consumption by the inhibition of cytochrome c oxidase (Chen et al., 2008; Cooper, 2002; Shiva et al., 2001). NO has proven benefit to cardiovascular health given that it can curb inflammation and oxidative stress under physiological conditions (Sharma et al., 2007), whereas inflammation promotes development of atherosclerosis (Ebenebe et al., 2020; Libby et al., 2002). Conversely, NO inhibits platelet aggregation, thereby limiting blood clot formation that can occlude blood vessels, a precursor to heart attacks and strokes (Nong et al., 1997). The inherent function of NO in cardiovascular biology is the promotion of blood flow and limiting CVD risk such as atherosclerosis and thrombosis at bay (Chen et al., 2008; Moncada et al., 2006). Consequently, a shortfall in NO synthesis is linked to CVDs (Tidball and Wehling-Henricks, 2014).

In addition to the multifaceted roles of NO, it can post-translationally modify and modulate cardiac proteins including kinases via *S-*nitrosylation (Broillet, 1999; Sun and Murphy, 2010; Foster et al., 2009). *S-*nitrosylation, a major effector of NO signaling, involves the addition of a NO group to the cysteine thiol (-SH) residues in proteins and the balance of *S*-nitrosylation is controlled by enzymes that metabolize *S*-nitrosothiols, for instance, *S*-nitrosoglutathione reductase (GSNO-R), which catalyzes the reduction of *S*-nitrosoglutathione (GSNO) (Irie et al., 2015). This modification causes conformational changes in proteins, can increase or decrease catalytic activity and induce complex formation. Protein *S*-nitrosylation has been regarded as transient signaling mechanism. *S*-nitrosothiols are labile and can transition to form disulfides by reacting with thiols. Even though there are exceptions where nitrosothiols that form on proteins do not exhibit the predicted high degree of reactivity. The stabilization of nitrosothiols in this case is conferred by the shielding of the nitrosothiol from cytosolic reducing agents through conformational changes (Paige et al., 2008). These disulfides are more stable and dominate over *S*-nitrosothiols after exogenous or endogenous nitrosative signaling. The overlap between *S*-nitrosylation and disulfides underscores a regulatory mechanism wherein *S*-nitrosylation transiently modulates protein function, potentially acting as a protective modification against irreversible oxidation, before transitioning to a disulfide state (Wolhuter et al., 2018; Lim et al., 2008).

**FIGURE 1:**
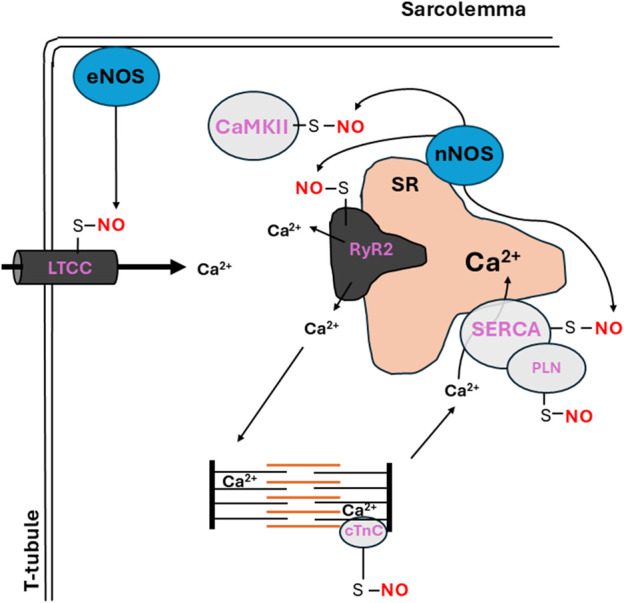
Overview of protein *S*-nitrosylation of Ca^2+^-handling proteins in the cardiomyocyte. RyR2 (ryanodine receptor 2) is located on the sarcoplasmic reticulum (SR) membrane near the L-type calcium channel (LTCC) through which Ca^2+^ enters and triggers further Ca^2+^ release from the SR into the cytosol via RyR2. The rise in Ca^2+^ leads to activation of Ca^2+^/Calmodulin-dependent protein kinase II (CaMKII) which can phosphorylate LTCC and RyR2 to promote Ca^2+^ release. Transient increase in Ca^2+^ initiates contraction at the myofilaments. When Ca^2+^ is removed from the cytosol, relaxation occurs. The Sarcoendoplasmic Reticulum Ca^2+^-ATPase (SERCA) then pumps Ca^2+^ back into the SR to restore Ca^2+^ stores. On the SR, neuronal nitric oxide synthase (nNOS) promotes *S*-nitrosylation of RyR2 and enhances Ca^2+^ release, while *S*-nitrosylation of CaMKII reduces its activity in the absence of β-adrenergic receptor stimulation. Endothelial Nitric Oxide Synthase (eNOS) regulates the *S*-nitrosylation of LTCC leading to reduction in channel activity and Ca^2+^ entry, which is a protective mechanism against ischemia/reperfusion injury. Myofilament protein, cTnC can also undergo *S*-nitrosylation, potentially influencing myocardial Ca^2+^ sensitivity via modulation of cross-bridge cycling. Meanwhile *S*-nitrosylation of phospholamban (PLN) diminishes the inhibitory effect on SERCA, increasing Ca^2+^ uptake into the SR.

Direct regulation of Ca^2+^-handling proteins can occur through *S-*nitrosylation with resultant effects being protein-, duration/degree- or site-dependent (Irie et al., 2015; Alim et al., 2022; Lima et al., 2010). In cardiomyocytes, S-nitrosylation regulates ECC by mediating the activity of Ca^2+^-handling proteins (Figure 1).

*S-*nitrosylation of RyR2 can increase RyR2 activity and hypo-nitrosylation of RyR2 increases Ca^2+^ leak and arrhythmogenesis in cardiomyocytes, suggesting the critical role of NO in cardiac contractility (Vielma et al., 2016; Gonzalez et al., 2007; Wang et al., 2010; Marx et al., 2001; Gonzalez et al., 2007). In heart failure, there is reduced NO bioavailability in turn there is an increase in oxidative stress, this redox modifications of RyR2 impairs the *S*-nitrosylation of the cysteine residues (Nikolaienko et al., 2018). This deficiency in RyR2 *S*-nitrosylation leads to diastolic Ca^2+^ leak (Gonzalez et al., 2010). Loss of NO availability which is associated with nitroso-redox imbalance promotes arrhythmic phenotype through increase in ROS. There is also an observed crosstalk between *S*-nitrosylation and phosphorylation as observed in the downregulation of RyR2 phosphorylation at S2814 (Burger et al., 2009; Cutler et al., 2012).

In LTCCs, *S-*nitrosylation via NOS modulates Ca^2+^ influx, particularly in response to β-adrenergic stimulation. During sympathetic activation as observed in I/R, β-adrenergic signaling enhances LTCC activity via protein kinase A (PKA)-mediated phosphorylation, leading to increased Ca^2+^ entry and enhanced cardiac contractility. However, *S*-nitrosylation of LTCC acts as a countereffect by decreasing channel activity, limiting SR Ca^2+^ at the beginning of ischemia, thereby reducing Ca^2+^ overload in early reperfusion and subsequently preventing I/R injury (Vielma et al., 2016; Sun et al., 2006).

*S-*nitrosylation of SERCA2a in cardiomyocytes after treatment with a NO donor, *S*-nitrosoglutathione (GSNO) enhances SR Ca^2+^ uptake into the SR and reduces Ca^2+^ overload during I/R (Sun et al., 2007). Meanwhile, *S*-nitrosylation of PLB diminishes its inhibitory effect on SERCA2a, leading to enhanced Ca^2+^ uptake into the SR. This modification facilitates efficient Ca^2+^ cycling and contributes to improved cardiac contractility (Irie et al., 2015; Vielma et al., 2016). Thus, there may be an interplay between PLB *S*-nitrosylation and SERCA2a activity, which may explain a critical mechanism by which NO signaling modulates cardiac function.

β-adrenergic stimulation has also been shown to induce *S*-nitrosylation of myofilament proteins (Irie et al., 2015). For instance, *S*-nitrosylation of cardiac troponin C at C84 in wild-type (WT) cardiomyocytes decreased myocardial Ca^2+^ sensitivity compared to GSNOR-tg cardiomyocytes. Notably, phosphorylation of cardiac troponin I (cTnI) at Ser22/23, a key determinant of Ca^2+^ sensitivity, was similarly enhanced by isoproterenol (ISO) in both WT and GSNOR-tg hearts. These findings indicate that *S*-nitrosylation may play a role in mediating the ISO-induced reduction in myofilament Ca^2+^ sensitivity, parallel to cTnI phosphorylation.

One kinase that has been implicated in cardiac diseases is CaMKII. Experiments have further demonstrated that the inhibition of CaMKII restores contractility and relaxation in the isolated hearts of type 2 diabetic rat models (Daniels et al., 2018), suppresses arrhythmic events in type 1 diabetic mouse myocytes (Hegyi et al., 2021), and promotes post-ischemic recovery in isolated hearts (Bell et al., 2014; Yao et al., 2022).

**FIGURE 2:**
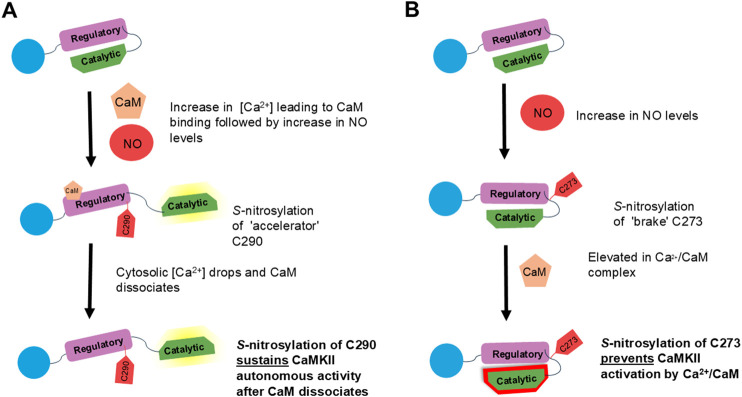
Overview of CaMKII *S*-nitrosylation sites and dual regulation of CaMKII activity in the heart. **(A)** Elevated [Ca^2+^] with CaM before increase in NO level allows access to C290 in the regulatory domain and *S*-nitrosylation of the C290 residue of CaMKII. This activates autonomous activity of the kinase even after [Ca^2+^] decreases and CaM dissociates. **(B)** Increase in NO levels before Ca^2+^/CaM results allows *S*-nitrosylation of the C273 residue that limits the ability of Ca^2+^/CaM to activate CaMKII. CaMKII, Ca^2+^/Calmodulin-dependent protein kinase II; NO, nitric oxide; CaM, Calmodulin.

CaMKII can regulate cardiac proteins and function by its direct phosphorylation of proteins such as RyR2, PLN and LTCC (Camors and Valdivia, 2014; Wehrens et al., 2004; Witcher et al., 1991; Zhang et al., 2003), but its activity can be modulated by the *S-*nitrosylation (Alim et al., 2022; Curran et al., 2014; Gutierrez et al., 2013; Coultrap and Bayer, 2014; Pereira et al., 2015; Erickson et al., 2015; Power et al., 2023). Recent evidence has shown that during stress response, there is an increase in NO production which can activate one of the cardiac-specific isoforms of CaMKII, δ, through *S-*nitrosylation and activation of this kinase via this mechanism can have protective or detrimental effects on cardiac function depending on the cysteine residue that is modified. In the heart, CaMKIIδ can be *S-*nitrosylated via two cysteine residues that alter function, Cys 273 and Cys 290. The Cys 273 site inhibits activation by Calcium/Calmodulin (Ca/CaM), while the Cys 290 site enhances autonomous activity. Studies have shown that treating cardiomyocytes with a NO donor before CaMKIIδ activation causes *S-*nitrosylation of the Cys 273 site and that prevents the increase in Ca^2+^ spark frequency due to β-adrenergic receptor (β-AR) activation. Conversely, when activation occurs before treatment with a NO donor, *S-*nitrosylation of the Cys 290 site promoted CaMKIIδ activity (Erickson et al., 2015) (Figure 2). Other studies have also shown that CaMKII contributes to pro-arrhythmic signaling in cardiomyocytes through *S-*nitrosylation even in the absence of β-adrenergic stress (Curran et al., 2014; Gutierrez et al., 2013). In addition, we have also demonstrated that CaMKIIδ *S-*nitrosylation can prevent or promote arrhythmias in isolated hearts depending on the order of β-adrenergic stimulation and NO treatment (Power et al., 2023). These findings further solidify the evidence that *S*-nitrosylation of CaMKIIδ plays a dual role in cardiac function. According to (Erickson et al., 2015), this dual role was attributed to the conformational change that occurs during *S*-nitrosylation before and after CaMKII activation. They hypothesized that the possible steric occlusion of the CaM binding site on the regulatory domain when the Cys 273 site is *S*-nitrosylated prevents CaM binding, thereby reducing CaMKII activity.

There is an interplay between PTMs, especially *S*-nitrosylation and oxidation since a balance between the two PTMs play a role in cardiovascular signaling. CaMKII oxidation (Erickson et al., 2008) has been found to contribute to cardiac dysfunction due to increase in ROS, especially in heart failure, arrhythmias and ischemic heart disease. Previous study observed that the C290 *S*-nitrosylation site also modulates oxidation-dependent CaMKII activity in the heart, therefore in pathological signaling such as I-R where there is increase in ROS, both oxidation and *S*-nitrosylation could be competing for this site. It is possible that this site alters the structural conformation of CaMKIIδ in a way that influences its redox sensitivity (Erickson et al., 2015).

While there is conflict on the exact role of *S-*nitrosylation on some cardiac proteins, the evidence of its deficiency being implicated in disease speaks strongly of its position as a stakeholder in cardiovascular health. One clear instance is where *S-*nitrosylation of protein kinase G (PKG) drives increased PKG activity, in turn, leading to reduced afterload due to vasodilation (Mangmool et al., 2023). In the heart, *S-*nitrosylation has been shown to work in tandem with phosphorylation to regulate and tune many cardiac Ca^2+^-handling proteins, thereby expanding its role in cardiac Ca^2+^ homeostasis (Irie et al., 2015). *S*-nitrosylation of cardiac CaMKII has been demonstrated to boost cardiac contractility acutely following a mechanical afterload (e.g., aortic valvular resistance and elevated arterial pressure) (Alim et al., 2022; Power et al., 2023; Reil et al., 2020). However, excessive *S*-nitrosylation of some cardiac proteins could be harmful to cardiac function depending on the degree of modification and duration. This then places *S*‐nitrosylation of cardiac proteins in a context where they may be specifically leveraged as a novel therapeutic to enhance myocardial function.

### 1.5 Sex differences in *S*-nitrosylation signaling

Sex hormones play a key role in NOS expression in the heart, which has previously been used as an explanation of sex differences in CVD-associated mortality (Hodgin et al., 2002). Estrogen has been shown to increase both eNOS and nNOS expression in murine studies which was concluded to be cardioprotective (Chen et al., 2003; Haynes et al., 2003; Shao et al., 2017). However, testosterone has only been shown to increase eNOS in vascular smooth muscle which rationalizes males being prone to hypertension at a younger age than females (Yu et al., 2010). In a human heart sample study, female samples were reported to have more NO in comparison to male samples (Schuh et al., 2017).

The effect of protein *S*-nitrosylation has been observed to be sex-dependent at the level of Ca^2+^ channels downstream to β_1_-AR and CaMKIIδ. A study on LTCC *S*-nitrosylation in mouse hearts reported that ISO induced female mouse hearts had significantly greater *S*-nitrosylated L-type Ca^2+^ channels than males (Sun et al., 2006). Furthermore, as estrogen has been established to decrease L-type Ca^2+^ channel expression and current (Ma et al., 2009), this increase in *S*-nitrosylation suggests that estrogen-NO signaling plays a key role in modulating the Ca^2+^ transients in conditions of acute β-adrenergic stress. The reduction in *S*-nitrosylation of male L-type Ca^2+^ channels therefore shows that lower levels/absence of estrogen would support greater Ca^2+^ transients to facilitate better cardiac function as the functional result of a superior β-adrenergic transduction pathway in young males when compared to young females (Parks and Howlett, 2013; Sun et al., 2006; Ma et al., 2009). It is also possible that higher I_Ca,L_ may facilitate excessive Ca^2+^ flux seen in heart failure (Gorski et al., 2015).

To further explain the role of estrogen in cardiac function, there are two key nuclear estrogen receptors (ER) in the body. The alpha subtype (ER-α) is associated with the pathophysiology of breast cancer. Whereas the beta subtype (ER-β) is associated with cardiac protection observed in females of childbearing age (Lin et al., 2009). A study (Lin et al., 2009) elucidated an interaction between ER-β and NO that contributed to cardiac protection. They reported that stimulation of ER-β increases NO production and facilitates two protective outcomes; 1) *S*-nitrosylation of cardiac enzymes to change their activity by inducing a structural conformational change, and 2) *S-*nitrosylation of cystine residues to protect them from being damaged by oxidation. These two protective outcomes are supported by several studies (Sun et al., 2006; Lin et al., 2009) and is in accordance with a study (Shao et al., 2016) reporting that *S*-nitrosylation of cardiac proteins occurs in a sex dependent manner. They reported the number of *S*-nitrosylated proteins to be higher at baseline in female mice hearts compared to males and this was associated with significantly increased eNOS expression and eNOS phosphorylation in females compared to males. Furthermore, increased *S-*nitrosylation of the mitochondrial compartments of female cardiomyocytes was observed and concluded to be in support of the second protective outcome against oxidative stress as suggested in a previous study (Lin et al., 2009). Together, these studies implicate a possible signaling mechanism that protects female hearts from developing CVD before menopause. We suggest this mechanism may be associated with female sex hormones signaling through ER-β to increase NOS expression and NO production, and thus *S*-nitrosylate cardiac enzymes to regulate their activity in the cardiac cycle, and *S-*nitrosylate available cysteines to protect them from the permanent damage of oxidative stress.

Taken together with CVD prevalence in males occurring earlier than females, this is consistent with NO production and signaling being cardioprotective and augmented via estrogen. Furthermore, a study reported that isoproterenol-induced female mouse heart had significantly greater *S-*nitrosylated proteins than males (Shao et al., 2016; Sun et al., 2006). This was shown to be specifically significant in *S-*nitrosylation of the LTCCs. As earlier mentioned that estrogen has been established to decrease LTCC expression and current (Ma et al., 2009), this increase in *S-*nitrosylation suggests NO signaling plays a key role in upregulating the Ca^2+^ transients in conditions of acute β-adrenergic stress, when under the influence of estrogen. This would support greater Ca^2+^ transient dynamic range of cardiac function increase as the result of β-adrenergic transduction pathway (Sun et al., 2006; Ma et al., 2009).

### 1.6 Is there a potential sex difference in CaMKII *S-*nitrosylation?

As CaMKIIδ is a key cardiac enzyme dually regulated by *S-*nitrosylation (Erickson et al., 2015), it is interesting to note that estrogen has also been established to both inhibit CaMKIIδ activity indirectly by inhibiting upstream receptor β_1_-AR and directly inhibit autonomous activation of CaMKIIδ (Ma et al., 2009). The two protective outcomes outlined previously (Lin et al., 2009) do fit regulation characteristics of CaMKIIδ during S-nitrosylation. 1) *S-*nitrosylation of the C273 site reduces activity by stabilizing autoinhibition and *S-*nitrosylation of the C290 increases activity by inducing autonomous activity; and 2) *S-*nitrosylation of C273 would protect it from being damaged by oxidation and no longer able to induce autoinhibition and prevent pathological autonomous activity (Erickson et al., 2015; Ma et al., 2009).

It is currently unknown if CaMKII *S*-nitrosylation differs in males and females, considering that females have been shown to have a higher higher NO availability following β-AR stimulation (Sun et al., 2006). With estrogen enhancing NO signaling to increase Ca^2+^ signaling, it is possible that the presence or absence of estrogen influences the level of CaMKII activity. Thus, we can postulate that *S-*nitrosylation of CaMKII facilitates an increase in phosphorylation of Ca^2+^-handling channels in a sexually dimorphic manner. Since *S-*nitrosylation of CaMKII influences autonomous activity, it is plausible that this modification is modulated by estrogen signaling in females. The absence of estrogen signaling in males may therefore reduce the amount of baseline CaMKII that is unavailable for activation (less *S*-nitrosylation at C273) allowing greater CaMKII signaling in the β-adrenergic transduction pathway (and conversely less in females). In response to acute stress, this is observed as a stronger functional increase in cardiac function. However, in chronic β-adrenergic conditions, it may be pivotal in the development of persistent cardiac dysfunction at an earlier age in males.

## 2 Conclusion

Ca^2+^ remains a key regulator of cardiac function, and cardiac proteins play crucial roles in maintain Ca^2+^ homeostasis. In pathological conditions such as arrhythmias and heart failure, dysregulation of Ca^2+^ cycling can occur due to PTMs such as phosphorylation, O-GlcNAcylation, oxidation and *S*-nitrosylation. Here, we have discussed the role of *S-*nitrosylation in cardiovascular functioning, dysfunction, and protection. Overall, protein *S-*nitrosylation and sex differences can be leveraged as potential novel biomarkers for early diagnosis of disease onset and progression. Furthermore, their therapeutic potential can be exploited in the context of individual health conditions, possibly placing them in the landscape of personalized medicine. Such sex differences and *S-*nitrosylation may be manipulated to the point of enhancing the safety and efficacy of some known cardioprotective strategies. It is already known that some PTMs may facilitate pre-and post-conditioning protection, hence, moving this towards patient care might be worth exploring (Dezfulian et al., 2013). Succinctly put, sex differences and protein *S-*nitrosylation, amongst other players, may reveal untapped and unexplored pathways and mechanisms for cardioprotection which may not yet be fully understood. It is currently unknown if sex influences *S-*nitrosylation of CaMKII in persistent cardiac dysfunction. However, as estrogen has been established to both inhibit CaMKII activity and autonomous activity (Ma et al., 2009), as well as increase nNOS expression and NO production (Sun et al., 2006; Ma et al., 2009; Chen et al., 2003; Ma et al., 2009), we postulated that estrogen signaling may be involved in baseline regulation of *S-*nitrosylation at the C273 vs. C290 sites of CaMKII, providing protection in females against overactivation of CaMKII. The lack of estrogen-dominated signaling in males may shift the homeostatic *S*-nitrosylation toward C290 vs. C273 sites of CaMKIIδ, thereby promoting CaMKII autonomous activation. This would implicate C290 *S-*nitrosylation to also be more upregulated because of a lack of estrogen signaling during β_1_-adrenergic signaling and thus more persistent cardiac dysfunction at an earlier age in males than females. Investigation of this novel dynamic provides a promising window into the underlying biochemistry and physiology which may contribute to the sex-specific disparities in CVD morbidities and mortality.

One major challenge in decoding the physiological and pathological relevance of protein *S*-nitrosylation in the heart has been the lack of real-time and specific detection methods. Useful approaches, such as the biotin-switch technique (BST) (Jaffrey and Snyder, 2001; Forrester et al., 2007; Kohr et al., 2011), have been instrumental in identifying nitrosylated targets, but are limited by low specificity, low efficiency and high false positive rates (Liang et al., 2024). Another commonly adopted method is the use of mass spectrometry to identify the specific sites of SNO modification and it enhances detection sensitivity by detecting peptide segments (Kohr et al., 2011; Derakhshan et al., 2007; López‐Sánchez et al., 2014; Thompson et al., 2013). There are limitations in the use of this method such as the accuracy of mass spectrometry instruments, potential recognition errors from low-abundance peptide segments and the requirement for large protein samples. However, recent technological innovations have significantly advanced the field. For example, two-dimensional nitrosylated protein fingerprinting using poly (methyl methacrylate) (PMMA) microchip platforms offers enhanced resolution and quality output, enabling the separation and detection of distinct *S*-nitrosylated isoforms in human colon epithelial adenocarcinoma cells (HT-29) and AD transgenic mice brain tissues (Wang, 2012). More recently, mass spectrometry-compatible strategies such as iodoTMT switch assay (Qu et al., 2014), SNOTRAP (Yang et al., 2023), and other chemoselective ligation-based assays have revolutionized the detection of protein *S*-nitrosylation by providing site-specific and reproducible quantitative assessments of protein *S*-nitrosylation (Wang, 2012). Many modifications have also been done on BST and Mass spectrometry to reduce experimental cost, increase specificity, detection efficiency and sensitivity (Liang et al., 2024). Moreover, the ability to resolve nitrosylation patterns with greater precision has implications for understanding sex differences and age-related remodeling in cardiac function. These advanced technologies hold promise for refining our understanding of how *S*-nitrosylation contributes to both homeostasis and disease, particularly when integrated with omics identification methods.

On the cautionary side, sex differences and protein *S-*nitrosylation amongst other key players are still quite complex and their effects on cardiac function and stress response are context-dependent, may vary greatly depending on the degree of modification or stimulus, duration, species, age, sex, and co-morbidities of the subject. These need to be considered to enable the adoption of balanced personalized strategies towards relieving CVD burden globally and provide more evidence for acknowledgment of sex in clinical management of CVDs. Therefore, further research into these biochemical and physiological sex differences could inform sex-specific diagnostics to prevent the progression of CVD and the development of arrhythmogenesis. This would not only be imperative to combat the worldwide burden of CVD morbidity and associated mortality but also contribute to improving quality of life by influencing the development of therapeutic interventions that take sex into consideration (Casin and Kohr, 2020).
